# Chinese Character Processing in Visual Masking

**DOI:** 10.3389/fpsyg.2021.763705

**Published:** 2022-02-24

**Authors:** Juan Chen, Ye Zhang

**Affiliations:** ^1^Center for Cognition and Brain Disorders, The Affiliated Hospital of Hangzhou Normal University, Hangzhou, China; ^2^Deqing Hospital of Hangzhou Normal University, Hangzhou, China; ^3^Zhejiang Key Laboratory for Research in Assessment of Cognitive Impairments, Hangzhou, China

**Keywords:** visual masking, mask form, temporal sequence, masking effect, depth of processing

## Abstract

It has not been clarified if attention influences perception of targets in visual masking. Three forms of common masks (random pattern, para-/metacontrast, and four dots) were thus chosen in the present study and presented with character targets in three temporal sequences (forward, backward, and sandwiched mask or forward-backward mask combination). In order to pinpoint the level of processing where masking arises, character targets were varied in depth of processing from random arrangements of strokes up to real Chinese characters. The attentional influence was examined under perceptual discrimination and lexical decision tasks, respectively. The results revealed significant interactions among four factors (mask form, temporal sequence, depth of processing, and task). Identification of character targets in each form of mask sequence varied with task demand, with greater suppression in the perceptual discrimination task. These findings suggested that attentional demand can bias processing in favor of task-related information in visual masking. Variations in masking effects may be contributed by both attentional demand and spatio-temporal interaction.

## Introduction

In our daily lives, we are able to detect words in streaming changing scenes (Rousselet et al., 2002; Rossion et al., 2015; King et al., 2016; Mohsenzadeh et al., 2018; Grootswagers et al., 2019, 2020; Robinson et al., 2019). These rapidly changing scenes impose a time limit on target word processing or even have words invisible due to the preceding or following stimuli (masks). It is not elucidated to what extent a word has been processed and whether a word could hold on to task requirements resisting the masking.

There have been numerous studies investigating the cognitive mechanisms of this masking phenomenon. The phenomenon is attributed to spatio-temporal dynamics of the visual system (Turvey, 1973; Jordan and de Bruijn, 1993). Different forms of masking, therefore, have been identified in terms of their spatio-temporal relations with targets. Three forms of spatial masks (pattern, para/meta-contrasts, and four dots) are mostly used in visual research. The former one spatially overlaps and shares structural features with the target, the latter two do not overlap with the target (Davis and Kim, 2011; Bachmann and Francis, 2013). In a stimulus sequence, the mask either precedes or follows the target, producing forward or backward masking (FM or BM) (Lleras and Moore, 2003). In some specific paradigms (rapid stream stimulation or RSS, see Rudell, 1992; Luo et al., 2019, 2021), the target occasionally appears in sequentially presented masks, such as both forward and backward masking. Two studies made systematical comparisons among three forms of backward masking (Enns, 2004; Chakravarthi and Cavanagh, 2009). They revealed that all forms of backward masks produced equal effects on the target identification at certain intervals (e.g., noise pattern and metacontrast masking at an interval of 50 ms and four-dot masking at intervals longer than 150 ms).

In explaining the masking phenomenon, theoretical models concern the level of processing where a specific form of masking exerts its influence. Earlier studies of pattern masking considered it reflecting the spatio-temporal integration of the target and the mask or interruption to the target processing at the low level (Schiller and Smith, 1965; Turvey, 1973; Jordan and de Bruijn, 1993). Metacontrast masking is emphasized to produce lateral inhibitory interactions between neurons that respond to the spatio-temporal properties of vision (e.g., metacontrast, Francis, 1997; Macknik and Livingstone, 1998). However, there were a few that suggested that contribution of higher-level processes, such as re-entrant processes in the metacontrast masking (Haynes et al., 2005; Fahrenfort et al., 2007). Enns and his colleagues emphasized that four-dot masking occurred at a later level of object processing if onsets of the target and four dots were simultaneous with the four-dots remaining on the view for a while after the target offset (Enns and Di Lollo, 1997, 2000).

Despite decades of research, few studies have clarified if attention affects the level of target processing in masking. In a study by Shelley-Tremblay and Mack (1999), the role of visual attention in masking was examined. They showed that meaningful stimuli (e.g., one’s name or a happy-face) greatly reduced the effectiveness of metacontrast backward masking when they were targets but increased the strength of masking when they served as masks. Enns (2004) found that spatial precueing of target letter location prior to the target-mask sequence minimized all forms of backward masking. Bruchmann et al. (2011) observed metacontrast facilitated target processing when targets were expected to appear at 100 ms after the cue onset, but not when targets were expected to appear at a longer interval (1,000 ms). A recent neuroimaging study by Grootswagers et al. (2021) revealed that attending to a visual stimulus (an object or a letter) as a task required enhancing the neural representations of the corresponding stimulus. However, Agaoglu et al. (2016) found that the attentional load indexed by set size does affect the performance of observers but does not interact with metacontrast masking when they asked observers to report the orientation of a masked target bar when presented with other randomly tilted distractor bars. Given these findings, to determine at which level of processing masking takes place, the influence of attention to masking needs to be further examined to clarify the variable findings among studies.

In this regard, the present study aimed at investigating the influence of attention in the perception of masked character targets in combination with other factors of spatio-temporal characteristic and depth of processing. The three forms of common masks (random pattern, para-/metacontrast, and four dots) were adopted, which were presented in three temporal sequences (forward, backward, and sandwich mask or forward-backward mask combination). To pinpoint the level of processing where masking arises, character targets were varied in the depth of processing from random arrangements of strokes up to real characters, indexed by character-likeness (non-character, pseudo-character, and real character). The attentional influence was examined under two kinds of cognitive tasks: perceptual discrimination and lexical decision. Derived from this design, we would like to observe interactions among four factors (mask form, temporal sequence, character-likeness, and task demand). This would suggest that the target identification in each form of mask sequence depends on the task demand, in particular, identification performance of each level of target character in masking varied with task demand with greater suppression in the perceptual discrimination task due to the task-orientated attention more focused on visual analysis of the target. Otherwise, task demand would be independent of the masked target processing, which would be demonstrated by similar identification performances of each level of character between tasks. Moreover, we expect to observe the variation of masking effectiveness of each mask form with temporal sequences for each level of target characters.

## Materials and Methods

### Participants

In this study, 37 undergraduate students from Hangzhou Normal University of China (9 men; range = 21–25 years old) were paid for participating in Experiment 1, and 30 students for Experiment 2 (7 men; range = 19–23 years old). All participants were right-handed with normal or corrected-to-normal vision. All were native Chinese speakers. Participants were provided informed consent, as approved by the institutional ethics committee of the Center for Cognition and Brain Disorders in Hangzhou Normal University.

**FIGURE 1:**
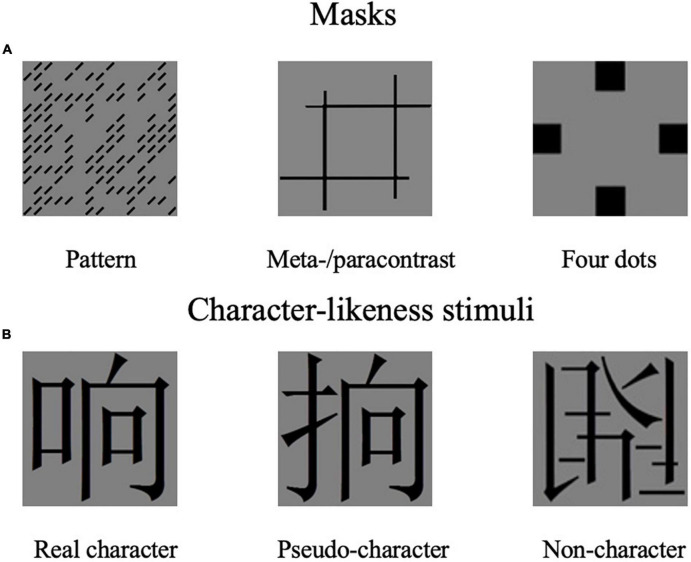
Masks and character-likeness stimuli in the two experiments.

Experiments 1 and 2 adopted the same materials. There were three forms of masks in the study (as shown in Figure 1A): patterns, metacontrast/paracontrast, and four dots. Pattern masking was random pattern of black oblique bars (each 12 × 2 mm in size) on the transparent background, used in the previous study (Oyama et al., 1983), containing 112 bars placed in positions randomly sampled with a probability of 0.5 from a 15 × 15 matrix (14 × 14 cm, 10 × 10 degrees). Half of the bars in the mask were oriented at 135 degrees, the other half were oriented at 45 degrees. Six different pattern masks for each bar orientation were used, as shown in Figure 1A. The metacontrast was four vertical and horizontal lines formed in two parallel pairs that are closely adjacent to the contours without superimposed with the Chinese characters. The width of lines used to construct the para/meta mask was 0.5 cm. The four-dot masking was modified with four squares surrounded by targets, and the square “dots” for a 2 cm × 2 cm.

Three hundred characters were chosen as target stimuli, whose orthography was manipulated to form character-like stimuli: real Chinese character (e.g., 响), orthographically correct pseudo-character (e.g., 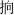), and stroke randomly combined non-character (e.g., 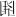) (as shown in Figure 1B). A hundred Chinese real characters were selected from the Modern Chinese Corpus Centre for Chinese Linguistics, Peking University^1^. All these real characters were left-right compound characters, with word frequency ranging from 877.7/million to 2,536/million (mean = 1,462.3, *SD* = 462.1). The stroke number of characters ranged from 7 to 11 (mean = 8.6, *SD* = 1.3). Pseudo-characters were made by combining radicals from two different characters according to orthographical rules but neither phonetic nor semantic components were present. Non-characters were constructed by combing the strokes of real characters randomly. There was a total of 100 pseudo-characters and non-characters. All the 300-character stimuli were presented randomly. All Chinese characters were Song font, black, and were presented on a gray background (RGB 128, 128, 128). The visual angle of stimuli was 4.4 × 4.4 degrees.

### Display

All stimuli were displayed on a 17-inch CRT (cathode-ray-tube) monitor (1,600 × 1,200 resolution) running at a refresh rate of 85 Hz by using JavaScript coded in the Visual Studio Code application. Participants were seated in a dimly lit room, attached to a chin rest at a distance of approximately 58 cm.

### Procedure

**FIGURE 2:**
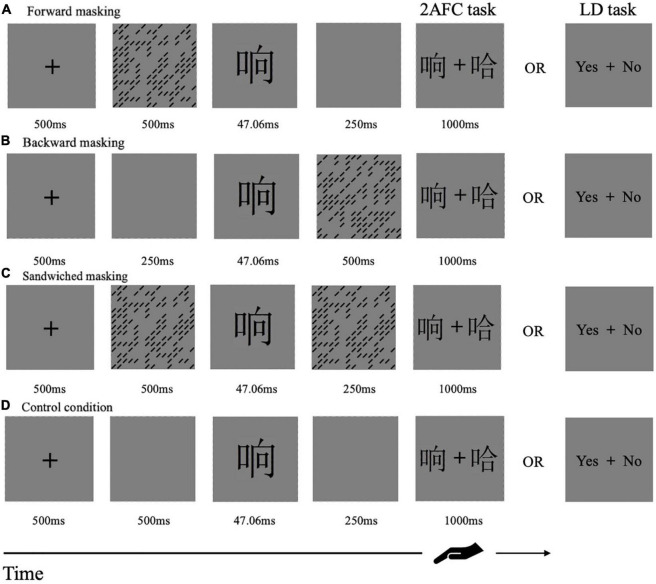
Schematic representation of the procedures of Experiment 1 and 2. Each row presented a single trial of stimulation sequences in the forward, backward, sandwiched masking, and control condition. The task was to decide which one was the target stimulus in Experiment 1 or whether the target stimulus was a real character or not in Experiment 2.

Each testing session began with several practice trials and was then followed by a formal experiment. Both Experiment 1 and 2 adopted 3 × 3 × 3 within-subject designs: mask form (patterns, metacontrast/paracontrast, and four-dot), character-likeness (real character, pseudo-character, and non-character), temporal sequence [forward (FM), backward (BM), and sandwiched masking (SM)]. The serial order of these manipulations was fully balanced across the observers in a Latin square. During the formal experiments, participants were asked to focus on the central fixation of a computer screen all the time. They were asked to respond as quickly and accurately as they could. Participants in Experiment 1 were asked to do a two-alternative forced choice task (2AFC task) and those in Experiment 2 had to finish a lexical decision task (LD task, which is to judge whether the target stimulus is a real character or not). Figure 2 showed experimental procedures in the two tasks. The position of the correct choice was randomly determined on each trial. The number of “yes” and “no” responses was balanced in each experiment. RTs and accuracies were recorded.

#### Forward Masking Condition

Each trial started with the central fixation point for 500 ms. Then, the mask was presented on the screen from 500 to 0 ms. After the presence of a target for 47.06 ms (4 screen refreshes), a blank screen was displayed for 250 ms. Two choices were followed, positioned left and right to the center of the fixation point. The response screen was presented for 1,000 ms (see Figure 2A).

#### Backward Masking Condition

Each trial started with the central fixation point of 500 ms. Then, the blank screen preceded the target presented by 250 ms. After the presence of the target for 47.06 ms, the mask was then followed for 500–0 ms. And the mask was immediately followed by the response screen (see Figure 2B).

#### Sandwiched Masking Condition

Each trial started with the central fixation point for 500 ms. Then, a mask was initially presented for 500 ms, and followed by a target for 47.06 ms. Another mask appeared backward for 250 ms. The backward mask was immediately followed by the response screen (see Figure 2C).

The threshold for 80% correct performance was determined by a one-up/three-down QUEST staircase procedure implemented using JavaScript (Watson and Pelli, 1983; Leek, 2001). If the correct response was made three times, then the duration of the mask was increased by 11.76 ms (one screen refresh). Trials continued until an error was made, in that case the duration of the mask for the next trial was shorter than 11.76 ms. In the SM condition, the initial display time for the mask was 500 ms, and the duration of the target was constant at 47.06 ms (4 screen refreshes). The duration of a second mask was 250 ms in the SM condition, with a variable duration of the preceding mask. The staircase ran for each block total of 108 reversals (turning points), and the threshold was the average of the last 54 reversals. There were four blocks, three blocks for three temporal sequences and one for an unmasked condition as control (see Figure 2D), and each block consisted of 360 trials. The increments of the 120 trials increased as the number of turning points until it reached 54 reversals.

### Analysis

The current analyses included three dependent variables. First, mean RTs for all conditions from correct trials were computed and analyzed for each subject. Second, instead of accuracy, a bias-free sensitivities index (A′) was calculated with the below equation (1) for all conditions. A′ is a non-parametric measure of sensitivity according to the signal detection theory and is relatively unaffected by response bias when the assumption of normality and equal variances are violated (Stanislaw and Todorov, 1999). It has been indicated that A′ is a better estimation of performance in a 2AFC task (Wong et al., 2012). The formula of A′ is shown below. Third, the magnitudes of masking effects indexed by ΔA′ and ΔRTs were calculated by subtracting the RTs and the A′s for choosing targets (real character, pseudo-characters, and non-characters) under each temporal sequence from those under the control condition(ΔA′ = control − A′; ΔRTs = control − RTs). A higher value of ΔRTs and ΔA′ designates a larger size of the masking effect, demonstrating bad performance, and relative disruption by masking.


(1) A=′0.5+[sign(H-F)(H-F)2+|H-F|4maX(H,F)-4HF]

*H* = hit rate, *F* = false alarm rate

A 3 × 3 ANOVA of mask form and character-likeness was conducted separately for each of the FM, BM, and SM conditions. *Post-hoc* comparisons were corrected with Bonferroni correction(Armstrong, 2014).

## Results

### Experiment 1

#### Forward Masking Condition

Different forms of masking reduced target sensitivities and partially delayed motor responses. The two-way ANOVA for A′ and mean RTs revealed significant main effects at mask form [A′: *F*(3, 108) = 5.494, *p* < 0.01, η_*p*_^2^ = 0.132; RTs: *F*(3, 108) = 139.170, *p* < 0.001, η_*p*_^2^ = 0.794] and character-likeness [A′: *F*(2, 72) = 11.840, *p* < 0.001, η_*p*_^2^ = 0.247; RTs: *F*(2, 72) = 98.700, *p* < 0.001, η_*p*_^2^ = 0.733]. Significant interaction was observed between mask form and character-likeness [A′: *F*(6, 216) = 2.406, *p* < 0.05, η_*p*_^2^ = 0.063; RTs: *F*(6, 216) = 60.046, *p* < 0.001, η_*p*_^2^ = 0.625]. *Post-hoc* comparisons of A′ and RTs for the mask form by character-like stimulus interaction when compared with the control condition are displayed in Table 1.

**TABLE 1:** The *p* values of simple effects in A′ and RT on masked characters across three temporal sequences in the 2AFC task.

	Real character			Pseudo-Character			Non-character		
2AFC	Pattern	Meta/Para-contrast	Four-dot	Pattern	Meta/Para-contrast	Four-dot	Pattern	Meta/Para-contrast	Four-dot
**A′**									
FM	0.226	0.211	0.000***	0.304	0.930	0.494	0.304	0.696	0.312
BM	0.000***	0.011*	0.011*	0.001**	0.000***	0.843	0.004**	0.368	0.016*
SM	0.345	0.000***	0.866	0.931	0.189	0.052	0.461	0.749	0.085
**RT**									
FM	0.159	0.000***	0.000***	0.000***	0.002**	0.000***	0.000***	0.000***	0.000***
BM	0.000***	0.241	0.000***	0.000***	0.000***	0.000***	0.000***	0.000***	0.000***
SM	0.013*	0.794	0.002**	0.000***	0.001**	0.001**	0.000***	0.000***	0.000***

*p < 0.05, **p < 0.01, and ***p < 0.001.

#### Backward Masking Condition

The results of A′ and mean RTs showed the significant main effects of character-likeness [A′: *F*(2, 72) = 7.182, *p* < 0.01, η_*p*_^2^ = 0.166; RTs: *F*(2, 72) = 40.163, *p* < 0.001, η_*p*_^2^ = 0.527] and mask form [A′: *F*(3, 108) = 12.526, *p* < 0.001, η_*p*_^2^ = 0.258; RTs: *F*(3, 108) = 152.946, *p* < 0.001, η_*p*_^2^ = 0.809] and significant mask form by character -likeness interaction [A′: *F*(6, 216) = 4.492, *p* < 0.001, η_*p*_^2^ = 0.111; RTs: *F*(6, 216) = 25.510, *p* < 0.001, η_*p*_^2^ = 0.415]. *Post-hoc* comparisons of A′ and RTs for the mask form by character-likeness interaction as compared with those in the control condition and are shown in Table 1.

#### Sandwiched Masking Condition

The results of the two-way repeated measures ANOVA for A*′s* and mean RTs revealed a significant main effect of character-likeness [A′: *F*(2, 72) = 21.127, *p* < 0.001, η_*p*_^2^ = 0.370; RTs: *F*(2, 72) = 330.084, *p* < 0.001, η_*p*_^2^ = 0.902, a significant main effect of mask form [A′: *F*(3, 108) = 13.633, *p* < 0.001, η_*p*_^2^ = 0.275; RTs: *F*(3, 108) = 92.171, *p* < 0.001, η_*p*_^2^ = 0.719], and an interaction between character-likeness and mask form [A′: *F*(6, 216) = 3.404, *p* < 0.01, η_*p*_^2^ = 0.086; RTs: *F*(6, 216) = 55.485, *p* < 0.01, η_*p*_^2^ = 0.606]. *Post-hoc* comparisons of A′ and RTs for the 2-way interaction are shown in Table 1.

**FIGURE 3:**
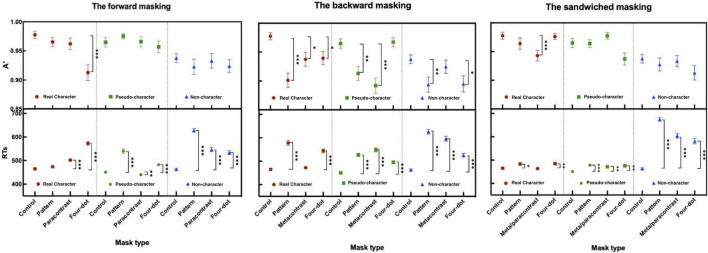
Sensitivity (A′) and mean RTs (ms) in the forward, backward, and sandwiched masking under the 2AFC task (the error bars indicate SE). *p < 0.05, ^**^p < 0.01, and ^***^p < 0.001.

All the significant comparisons in A′ and RTs in Experiment 1 are labeled in Figure 3.

### Experiment 2

#### Forward Masking Condition

The two-way ANOVA for A′ and mean RTs revealed significant main effects at character-likeness [A′: *F*(2, 58) = 31.943, *P* < 0.001, η_*p*_^2^ = 0.524; RTs: *F*(2, 58) = 50.442, *P* < 0.001, η_*p*_^2^ = 0.635], where identifying the pseudo-characters had the worst performance of all. The significant differences have shown main effects of mask form [A′: *F*(3, 87) = 8.496, *p* < 0.001, η_*p*_^2^ = 0.227; RTs: *F*(3, 87) = 6.322, *p* < 0.01, η_*p*_^2^ = 0.179] and mask form by character-likeness interaction [*F*(6, 174) = 4.157, *p* < 0.01 η_*p*_^2^ = 0.125; *F*(6, 174) = 3.427, *p* < 0.01, η_*p*_^2^ = 0.106]. *Post-hoc* comparisons of A′ and RTs showed significant differences relative to those in the control condition (refer to Table 2 for details).

**TABLE 2:** The *p* values of of simple effects in bias-free sensitivities index (A′) and RT on masked characters across three temporal sequences in the LD task.

	Real character			Pseudo-Character			Non-character		
LD	Pattern	Meta/Para-contrast	Four-dot	Pattern	Meta/Para-contrast	Four-dot	Pattern	Meta/Para-contrast	Four-dot
**A′**									
FM	.008**	0.026*	0.020*	0.508	0.978	0.577	0.001**	0.009**	0.033*
BM	0.000***	0.123	0.336	0.395	0.761	0.219	0.001**	0.001**	0.005**
SM	0.002**	0.075	0.547	0.152	0.326	0.943	0.002**	0.008**	0.034*
**RT**									
FM	0.206	0.218	0.781	0.135	0.696	0.259	0.000***	0.000***	0.000***
BM	0.004**	0.003**	0.032*	0.725	0.777	0.771	0.000***	0.000***	0.000***
SM	0.003**	0.756	0.847	0.011*	0.202	0.099	0.000***	0.000***	0.000***

*p < 0.05, **p < 0.01, and ***p < 0.001.

#### Backward Masking Condition

The results of A′ and mean RTs showed significant main effects at character-likeness [A′: *F*(2, 58) = 45.551, *P* < 0.001, η_*p*_^2^ = 0.611; RTs: *F*(2, 58) = 27.645, *P* < 0.001, η_*p*_^2^ = 0.488] and at mask form [A′: *F*(3, 87) = 17.295, *p* < 0.001, η_*p*_^2^ = 0.374; RTs: *F*(3, 87) = 15.676, *p* < 0.001, η_*p*_^2^ = 0.351] and a mask form by character-likeness interaction [A′: *F*(6, 174) = 6.656, *p* < 0.001, η_*p*_^2^ = 0.187; RTs: *F*(6, 174) = 11.294 *p* < 0.001, η_*p*_^2^ = 0.280]. *Post-hoc* comparisons of A′ and RTs revealed a significant masking effect when targets followed by as compared with the control condition (as shown in Table 2).

#### Sandwiched Masking Condition

The results of the two-way repeated measures ANOVA for A′ and mean RTs revealed that significant main effects of character-likeness [A′: *F*(2, 58) = 42.934, *p* < 0.001, η_*p*_^2^ = 0.597; RTs: *F*(2, 58) = 33.412, *p* < 0.001, η_*p*_^2^ = 0.535] and mask form [A′: *F*(3, 87) = 13.853, *p* < 0.001, η_*p*_^2^ = 0.323] and RTs: *F*(3, 87) = 13.706, *p* < 0.001, η_*p*_^2^ = 0.321]. A significant interaction was shown between character-likeness and mask form [*F*(6, 174) = 4.723, *p* < 0.001, η_*p*_^2^ = 0.140; *F*(6, 174) = 5.810, *p* < 0.001, η_*p*_^2^ = 0.167]. *Post-hoc* comparisons of A′ and RTs showed that all three forms of masks had a significant effect on non-characters (all *ps* < 0.05; as shown in Table 2).

**FIGURE 4:**
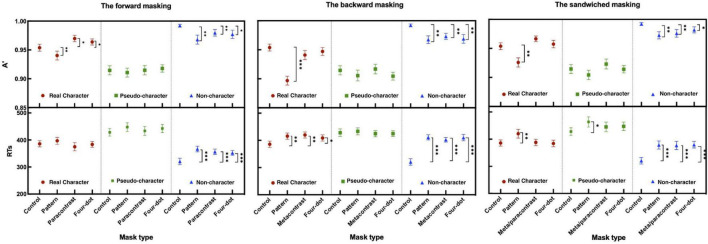
Sensitivity (A′) and mean RTs (ms) in the forward, backward, and sandwiched masking under the LD task (the error bars indicate SE). *p < 0.05, **p < 0.01, and ***p < 0.001.

All the significant comparisons in A′ and RTs in Experiment 2 are labeled in Figure 4.

### Task Demand Analyses

To test if the task modulates the masking effect on character processing, the task demand was taken as a between-group factor. A 4-way ANOVA of ΔA′ revealed a significant main effect of mask form [*F*(2, 130) = 8.351, *p* < 0.001, η_*p*_^2^ = 0.114]. Significant main effect of temporal sequence was observed [*F*(2, 130) = 23.754, *p* < 0.001, η_*p*_^2^ = 0.268]. There was a significant 4-way interaction [*F*(8, 520) = 4.154, *p* < 0.001, η_*p*_^2^ = 0.06]. *Post-hoc* analyses revealed that greater masking effects were observed on real characters in the paracontrast and four-dot FM under the 2AFC than those under the LD task, *t*(66) = 4.501, *p* < 0.05; *t*(66) = 18.905, *p* < 0.001, and in the metacontrast SM under the 2AFC task than those under the LD task, *t*(66) = 18.877, *p* < 0.001. Masking effects by pattern and metacontrast BM were stronger for pseudo-character under the 2AFC than the LD task, *t*(66) = 5.166, *p* < 0.05; *t*(66) = 13.196, *p* < 0.01. Results of *post-hoc* comparisons are displayed in Table 3.

**TABLE 3:** The *p* values for task effects in ΔA′ on masked characters in three temporal sequences.

	Real character			Pseudo-Character			Non-character		
2AFC vs. LD	Pattern	Meta/Para-contrast	Four-dot	Pattern	Meta/Para-contrast	Four-dot	Pattern	Meta/Para-contrast	Four-dot
**ΔA′**									
FM	0.909	0.038*	0.000***	0.250	0.953	0.419	0.568	0.575	0.942
BM	0.250	0.138	0.068	0.026*	0.001**	0.420	0.262	0.700	0.339
SM	0.345	0.000***	0.628	0.431	0.757	0.111	0.543	0.431	0.368
Δ**RT**									
FM	0.806	0.000***	0.000***	0.000***	0.209	0.164	0.000***	0.000***	0.004
BM	0.000***	0.027	0.000***	0.000***	0.000***	0.000***	0.000***	0.001**	0.084
SM	0.190	0.681	0.035	0.575	0.792	0.645	0.000***	0.000***	0.000***

*p < 0.05, **p < 0.01, and ***p < 0.001.

**FIGURE 5:**
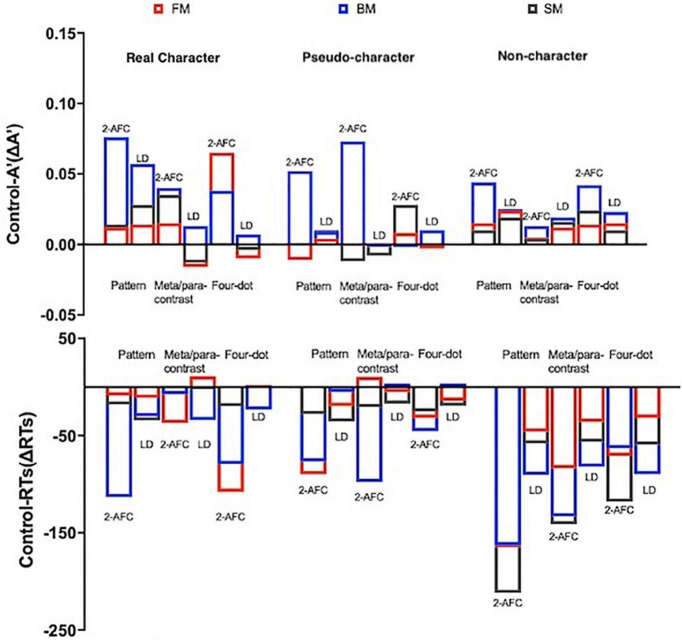
Difference of sensitivities (ΔA′) and ΔRTs between the two tasks (the error bars indicate SE). ΔA′ = control – A′, ΔRTs = control – RTs.

As for ΔRTs, there were significant main effects of character-likeness [*F*(2, 130) = 85.973, *p* < 0.001, η_*p*_^2^ = 0.569], mask form [*F*(2, 130) = 80.819, *p* < 0.001, η_*p*_^2^ = 0.554], temporal sequence [*F*(2, 130) = 16.573, *p* < 0.001, η_*p*_^2^ = 0.203]. A significant 4-way interaction was observed [*F*(8, 520) = 16.787, *p* < 0.001, η_*p*_^2^ = 0.205]. *A post-hoc* analysis for the interaction was conducted (as shown in Table 3 and Figure 5).

## Discussion

The present study aimed at investigating the influence of attention in the perception of masked character targets. The attentional influence to the masking effects was observed under different task requirements. The overall effectiveness of the masking of the targets under each task was demonstrated *via* variations in response sensitivity and reaction time. As expected, the results revealed a significant interaction among four factors, including mask form, temporal sequence, character-likeness, and task demand, suggesting identification of the masked targets modulated by task-orientated attention. The task requirement interacting with spatio-temporal properties of masks elicited different response pattern for three types of character targets.

### Attentional Modulation to Visual Masking

The task-oriented attention had their distinct influences on the target identification in all forms of temporal masking. The overall masking effects were stronger in backward masking under the 2AFC task, but greatly reduced or the target sensitivity was enhanced under the LD task. These task-related changes might reflect the attentional selection, which can bias the processing in favor of task-related features or objects. This is also evidenced by a previous study by Pilling and Gellatly (2010). They presented additional display items to influence the linking apparent motion seen between a target and a spatially separated mask and used placeholders to maintain the presence of target object during mask presentation, both manipulations resulted in a significant reduction of masking when the conditions promoted the target-mask individuation. It is suggested that attention involves not only the facilitation of needed information but also the suppression of unneeded information (Boyer and Ro, 2007; McMains and Kastner, 2009; Grootswagers et al., 2021). The unneeded stimuli (e.g., masks) or task-irrelevant information are decoded in the visual hierarchy, resulting from a degree of automaticity in visual processing (Grootswagers et al., 2019, 2021). This can explain why the masking across the tasks is active no matter how attention facilitates the target processing. Attention enhanced sensitivity of the targets in the masking by having the processing of the task-relevant information take priority over that of task-irrelevant one (Grootswagers et al., 2021). Moreover, we observed that the sensitivity enhancement of the targets in the masking was obvious in real characters and pseudo-characters under the LD task. The differences in task effect indicated that the magnitude of enhancement in sensitivity depends on the nature of the attended or task-relevant information. When attention was directed to lexical components under the LD task, processing was biased up to abstract properties of target characters and greatly suppresses the visual interference from masks. But when attention was directed to the perceptual contents under the 2AFC task, masking became more effective due to spatio-temporal alignments with the targets in the visual pathway.

Besides, Bachmann and Francis suggested that the target processing was facilitated mainly *via* conscious awareness (Bachmann and Francis, 2013). The conscious awareness happens because of the slower non-specific subcortical activity elicited by various mental preparations, such as pre-and post-target state or temporal expectancy modulates faster specific cortical activity generated by the target stimulus and participating in the representation of the target sensory-perceptual contents (Bachmann and Francis, 2013). In the case of the current experiment settings, as they suggested, awareness of the task requirements prior to the target-mask sequence or temporal expectancy induced by periodic stimulation might help upcoming targets upgraded to the conscious representation ahead. However, the task effects on target sensitivity in masking cannot be well-explained by this account. He et al. (1996) suggest that the availability of visual information to conscious awareness is restricted by the attention that acts in one or higher visual cortical areas. The differences in masking effectiveness between tasks are likely attributed to attentional focus on specific representation levels of the task-relevant information.

### Temporo-Spatial Interference to Target Processing

Our results revealed significant interactions between mask form and character-likeness across temporal sequences regardless of the task requirement. This suggested that the tempo-spatial influence on the target processing is unavoidable in visual masking. This influence was shown in all levels of processing. For instance, all forms of backward masks are observed to reduce the sensitivity of real characters under the 2AFC task and that of non-characters under the LD task, while pattern and metacontrast backward masking reduced sensitivity of pseudo-characters under the 2AFC task. The identification of real characters involves all levels of processing from visual analysis to lexical access, whereas non-characters and pseudo-characters well represent the featural and character form analysis, respectively. It seems that visual masking regardless of mask forms might involve either low or higher levels of processing, specific to characters corresponding to the feature analysis or lexical access. The masking effects on pseudo-characters by pattern and metacontrast might relate to their suppressions in the midlevel of character processing or character form analysis. It seems that these findings are compatible with the re-entrant account proposing that processing of visual characters in masking is a process of testing perceptual hypotheses through re-entrant iterative comparisons from the high-level areas to the ongoing low-level activity (Enns and Di Lollo, 2000). Alternatively, Chakravarthi and Cavanagh (2009) argued that all forms of backward masking lead to an abrupt attenuation of target signals at the earlier stage and thus result in the lack of later or higher-level feedback processing. Evidence has been shown that backward masking by noise and metacontrast have their maximally inhibitory effects on letter identification at a target-mask interval of 0–50 ms, whereas masking by four dots showed a similar effect at an interval later than 150 ms (Enns, 2004). The target-mask interval of 47 ms in our study is just at this interval when metacontrast and pattern masks maximize their spatial interference. The four-dot backward masking at our short interval indicated that its interference can occur as early as other forms of masking and influences the low level of processing. The masking at this level is associated with different mechanisms depending on the form of masks. The pattern masking works through spatio-temporal integration (Oyama et al., 1983; Enns, 2004), while the underlying mechanism of the metacontrast masking is transient-on-sustained inter-channel inhibition (Breitmeyer et al., 2006).

In contrast to the backward masking, forward masking by four dots and paracontrast had either facilitatory or inhibitory effects on target sensitivity across tasks in the current findings. These two forms of masks might have similar masking mechanisms. Kafaligönül et al. (2009) revealed that variations of paracontrast masking occur at different SOA ranges, modulated by the task requirement. The prolonged inhibition and the facilitation were explained as activities of slower cortical and subcortical systems (Breitmeyer et al., 2006; Kafaligönül et al., 2009). Breitmeyer (2007) suggested that paracontrast suppresses primarily the early feedforward processing and then indirectly weakens late re-entrant activity.

### Theoretical Implication

In closing, the present findings extend previous studies of this field by considering multiple interactions among factors, demonstrating contributions of spatio-temporal interaction and attentional demand in the identification of the masked target characters. Polat et al. (2007) have suggested that the masking effect depends on at least three factors: processing time of a target, temporal sequence, and spatial arrangement. Moreover, our findings might help to explain the variability of studies driven by differential attention mechanisms.

According to the recurrent processing model, visual recognition is a consequence of both feedforward and re-entrant processing, in which the visual input is transformed through cortical areas *via* feed-forward pathways and *via* re-entrant pathways from higher to lower areas (Enns and Di Lollo, 2000; Breitmeyer, 2007; Dux et al., 2010; Mohsenzadeh et al., 2018). Both forward and backward masking can cause suppression of early feedforward processing. This early suppression may be compensated by increased re-entrant processing at later stages (Mohsenzadeh et al., 2018). Future research should try to combine neuroimaging methods to clarify what are the contributions of cognitive processes (conscious awareness) supported through subcortical activities.

## Data Availability Statement

The original contributions presented in the study are included in the article, further inquiries can be found online in https://osf.io/d5g4j/?view_only=65ce940b83fe44019f77bc716fdfb00d.

## Ethics Statement

The studies involving human participants were reviewed and approved by the Hangzhou Normal University. The patients/participants provided their written informed consent to participate in this study.

## Author Contributions

YZ and JC contributed to the concept and designed of the experiments and wrote the manuscript. JC collected and analyzed the data. Both authors contributed to the article and approved the submitted version.

## Conflict of Interest

The authors declare that the research was conducted in the absence of any commercial or financial relationships that could be construed as a potential conflict of interest.

## Publisher’s Note

All claims expressed in this article are solely those of the authors and do not necessarily represent those of their affiliated organizations, or those of the publisher, the editors and the reviewers. Any product that may be evaluated in this article, or claim that may be made by its manufacturer, is not guaranteed or endorsed by the publisher.

## Footnotes

1
http://ccl.pku.edu.cn:8080/ccl_corpus/index.jsp?dir=xiandai
